# The Efficacy of Topical or Systemic Antibiotics as Adjuvants to Non-Surgical Periodontal Treatment in Diabetic Patients: A Systematic Review and Meta-Analysis of Randomized Clinical Trials

**DOI:** 10.3390/jcm13164763

**Published:** 2024-08-13

**Authors:** Rafael Scaf de Molon, Joao Victor Soares Rodrigues, Mariella Boaretti Deroide, Davi da Silva Barbirato, Valdir Gouveia Garcia, Leticia Helena Theodoro

**Affiliations:** 1 Department of Diagnosis and Surgery, School of Dentistry, São Paulo State University—UNESP, Aracatuba 16015-050, SP, Brazil; joao.vic.t@hotmail.com (J.V.S.R.); mariella_boaretti@hotmail.com (M.B.D.); vg.garcia@uol.com.br (V.G.G.); leticia.theodoro@unesp.br (L.H.T.); 2 Department of Basic and Oral Biology, Faculty of Dentistry of Ribeirão Preto, University of São Paulo (FORP/USP), Av. Café, S/N-Ribeirão Preto, São Paulo 14040-904, SP, Brazil; davibarbirato@gmail.com; 3 Latin American Institute of Dental Research and Teaching (ILAPEO), Curitiba 80710-150, PR, Brazil

**Keywords:** antibiotics, diabetes mellitus, periodontal disease, non-surgical periodontal treatment

## Abstract

**Background:** Periodontitis and diabetes mellitus (DM) exhibit a bidirectional relationship and are globally significant systemic chronic conditions. The utilization of antibiotics alongside non-surgical periodontal treatment (NSPT) has been a subject of investigation in numerous clinical studies involving human subjects. Thus, the objective of this systematic review is to address the following question: “What is the efficacy of scaling and root planing (SRP) associated with antimicrobials in patients with type 2 DM and periodontitis?”. **Methods:** A systematic review of the literature was conducted encompassing databases such as MEDLINE/PubMed, Scopus, and Web of Science up to July 2024. Additionally, alerts were configured to capture studies published from the initial search until manuscript submission. Randomized clinical trials assessing clinical periodontal parameters in DM patients undergoing SRP and receiving either topical or systemic antibiotics were compared against a control group (SRP only). Two investigators independently screened articles, extracted data, and evaluated their quality. The selection process, study characteristics, risk of bias, impact of antibiotics on clinical parameters, and certainty of evidence were elucidated in both textual and tabular formats. Meta-analysis was performed separately with forest plots generated for treatment modalities, period of evaluation, and type of antibiotics used. **Results:** Following the analysis of abstracts and full articles, a total of 30 randomized clinical trials were incorporated into this review, comprising 9 studies on the association of topical antibiotics and 21 studies on systemic antibiotic administration. The principal periodontal parameters assessed included probing pocket depth (PPD), clinical attachment level (CAL), plaque index (PI), and bleeding on probing (BoP). **Conclusions:** Analysis of the results led to the conclusion that adjunctive periodontal treatment with either topical or systemic antibiotics confers subtle clinical benefits. Nevertheless, owing to the heightened emergence of resistant bacteria and potential side effects, the use of antibiotic therapy in periodontal treatment should be judiciously administered.

## 1. Introduction

Diabetes mellitus (DM) is a chronic condition characterized by either partial or complete deficiency in insulin production or by resistance to its effects. This leads to disruptions in carbohydrate, protein, and lipid metabolism, resulting in hyperglycemia and triggering multiple systemic abnormalities [1]. By 2045, it is estimated that around 693 million individuals will be affected by DM, with approximately 90% of cases attributed to type 2 DM, characterized by insulin production alongside resistance to its actions [2]. The global prevalence of DM among adults has seen a significant rise, escalating from 4.7% in 1980 to 8.5% in 2014. Data from the World Health Organization indicate that approximately 451 million people worldwide were living with DM in 2017, and in 2019, diabetes was the cause of more than 1.5 million of deaths worldwide [3].

Periodontitis is a chronic inflammatory condition, influenced by multiple factors, associated with dysbiosis of the bacterial biofilm, and characterized by the gradual deterioration of the supporting periodontium, including cement, the periodontal ligament, and alveolar bone [4]. The severity of this inflammatory response is contingent upon the virulence of the microorganisms present and the susceptibility of the host, potentially resulting in damage to periodontal tissue [5]. In a consensus report of the workgroup of the 2017 World Workshop on the Classification of Periodontal and Peri-Implant Diseases and Conditions, periodontitis is now categorized according to the disease severity and complexity (stages 1 to 4) and rate of progression (grades A to C), making the diagnosis and treatment plan of periodontitis patients more accurate and reliable [4]. Of importance, the more severe forms of periodontitis have been implicated in aggravating the outcomes of other non-communicable chronic diseases such as cardiovascular disease [6,7], DM [8,9], pulmonary disease [10], rheumatoid arthritis [11,12], and others.

The bidirectional interplay between diabetes and periodontitis has garnered significant attention in the scientific communities over the last three decades. DM stands as one of the primary risk factors for periodontitis, with longitudinal and cross-sectional studies demonstrating that people with diabetes face a 3 to 4 times higher risk of developing severe periodontitis compared to non-diabetic patients [13]. Moreover, there exists a direct correlation between the degree of glycemic control and the severity of periodontitis [14,15]. Studies suggest that DM reduces collagen levels in periodontal tissues, thereby fostering increased tissue degradation [16,17]. Additionally, in individuals with DM, proteins undergo glycation, resulting in the formation of advanced glycation end products (AGEs), which elicit various effects via cellular interactions. These effects include alterations in macrophage function and the stimulation of the release of inflammatory mediators such as growth factors and cytokines, consequently prolonging and aggravating the inflammatory response [18,19,20].

Among the interventions for treating periodontitis, oral hygiene instructions, scaling and root planing (SRP) with or without adjunctive therapies such as systemic or topical antibiotics, the use of phytotherapies (herbal medicine) [21,22], specialized pro-resolving mediators [23], or even a combination of these procedures can be considered [24]. The “gold standard” for maintaining periodontal health is the disruption of dental biofilm through mechanical debridement. However, in cases involving deep pockets, furcation lesions, vertical bone defects, and/or teeth mobility, this approach may prove insufficient due to significant access and technical difficulties [24].

The use of antibiotics as adjunctives to periodontal therapy in non-surgical periodontal treatment (NSPT) can be either systemic or topical. Local/topical administration involves inserting antibiotics into the periodontal pocket. Due to its localized nature, this method reduces potential systemic side effects and ensures a higher concentration at the intended site without requiring patient compliance. Conversely, systemic antibiotics are typically administered orally, involving the ingestion of one or more pills. They offer broader coverage, albeit at lower concentrations, allowing them to reach the entire oral cavity.

The association of antibiotics with NSPT in diabetic patients has been under evaluation since the 1990s. Initial findings suggested that combining periodontal treatment with antibiotic therapy could assist in glycemic control in DM patients [25]. However, this topic remains a subject of debate, with numerous studies conducted to elucidate these findings, yielding some conflicting results [26,27,28]. Studies linking antibiotics with NSPT have demonstrated a favorable rate of clinical improvement, founded on the premise that this class of drugs serves as a significant ally in metabolic and periodontal pathogen control, thereby mitigating the inflammatory process and consequent damage to periodontal tissues [29].

However, the use of antibiotics in NSPT should not be indiscriminate, considering the emergence of resistant bacterial strains and potential side effects [30]. Hence, the objective of this study was to assess the effects of systemic and topical antibiotic administration as adjuncts to NSPT, facilitating an analysis of the outcomes and offering critical insights into antibiotic use in periodontal treatment for diabetic individuals with the aim of informing future research endeavors and treatment protocols.

## 2. Materials and Methods

### 2.1. Protocol Registration and PICO Strategy

The present systematic review was conducted in adherence to the guidelines established by the Cochrane Collaboration [31] and followed the principles outlined in the Preferred Reporting Items for Systematic Reviews and Meta-analysis (PRISMA) [32]. This systematic review was registered with the International Prospective Register of Systematic Reviews (PROSPERO) at the National Institute for Health Research (http://www.crd.york.ac.uk/PROSPERO), and it received approval under the register protocol number: CRD42021259515. The protocol assess date was started on 9 August 2021 and can be accessed at the following address: https://www.crd.york.ac.uk/prospero/display_record.php?ID=CRD42021259515. Moreover, the electronic search for the included articles started in 9 September 2021.

This study’s PICO question was formulated as follows: “What is the efficacy of SRP associated with antimicrobials in patients with type 2 DM and periodontitis?” The components of the PICO framework are as follows:

P (population)—patients diagnosed with any stage of periodontitis and type 2 DM;

I (intervention)—SRP (either conventional scaling or a whole-mouth approach) combined with adjunctive use of systemic or topical antibiotics; 

C (comparison)—patients diagnosed with type 2 DM and periodontitis who received treatment solely with NSPT without antibiotics; 

O (outcome)—the primary outcome considered were changes in clinical attachment level (CAL). Secondary outcomes included changes in bleeding on probing (BoP), probing pocket depth (PPD), and gingival index (GI). 

### 2.2. Search Strategy

This research study was conducted across three databases: MEDLINE/PubMed, Scopus, and Web of Science accessed on 6 March 2023. The search strategy was developed utilizing the controlled vocabulary “Medical Subject Headings” (MeSH). The keywords employed for the search were as follows: (“periodontal diseases” [MeSH Terms] OR “Periodontitis” [MeSH Terms] OR “chronic periodontitis” [MeSH Terms] OR “aggressive periodontitis” [MeSH Terms] OR “periodont*” [Title/Abstract]) AND (“glucose metabolism disorders” [MeSH Terms] OR “prediabetic state” [MeSH Terms] OR “diabetes mellitus” [MeSH Terms] OR “prediabet*” [Title/Abstract] OR “diabet*” [Title/Abstract]) AND (“therapy” [MeSH Subheading] OR “therapeutics” [MeSH Terms] OR “therap*” [Title/Abstract] OR “treat*” [Title/Abstract] OR “disease management” [Title/Abstract] OR “care” [Title/Abstract] OR “periodontal debridement” [MeSH Terms] OR “subgingival curettage” [MeSH Terms] OR “dental scaling” [MeSH Terms] OR “root planing” [MeSH Terms] OR “scaling and root planing” [Title/Abstract] OR “nonsurgical periodontal treatment” [Title/Abstract] OR “nonsurgical periodontal therapy” [Title/Abstract] OR “non-surgical periodontal treatment” [Title/Abstract] OR “non-surgical periodontal therapy” [Title/Abstract] OR “subgingival instrumentation” [Title/Abstract]) AND (“Anti-Infective Agents” [MeSH Terms] OR “Anti-Bacterial Agents” [MeSH Terms] OR “anti-microbial agents” [Title/Abstract] OR “anti-microbial agents” [Title/Abstract] OR “Antimicrobial Agents” [Title/Abstract] OR “Microbicides” [Title/Abstract] OR “Microbicide” [Title/Abstract] OR “Antibiotics” [Title/Abstract] OR “Antibiotic” [Title/Abstract]). This strategy was then tailored for each respective database (Scopus and Web of Science) and included the following key terms: periodontal disease, periodontitis, diabetes mellitus, clinical attachment level, antibiotics, antimicrobial agents, metabolic disease, non-surgical periodontal treatment, non-surgical periodontal therapy, periodontal treatment, subgingival instrumentation, scale and root planing.

### 2.3. Eligibility Criteria

The selection criteria for the studies encompassed randomized clinical trials that included patients diagnosed with both DM and periodontitis and studies that implemented NSPT in conjunction with antibiotic therapy (both topical and systemic). The exclusion criteria consisted of literature reviews, clinical case reports, pre-clinical studies, case series, studies lacking clinical periodontal data, and studies not published in English. Throughout the evaluation process, there were no discrepancies among the reviewers in the selection of studies. Dosage and route of administration were not considered exclusion criteria.

### 2.4. Selection Process

The articles retrieved were exported to Rayyan Reference Manager (https://www.rayyan.ai) and duplicates were eliminated through a combination of programmatic (perfect match) and manual methods, accessed on 5 February 2024. The selection procedure occurred in a dual-phase approach: Initially, two researchers (J.V.S.R. and M.B.D.) autonomously reviewed the titles and abstracts of all obtained references. Subsequently, the same two reviewers independently applied the exclusion criteria during the screening of full-text documents. The entire document was assessed and evaluated in its completeness. Cohen’s kappa was employed to establish inter-rater reliability in the process of study selection, with an acceptable threshold value of 0.80. Any disagreements at any stage were resolved through discussion and mutual agreement with a third reviewer (R.S.d.M).

### 2.5. Data Collection Process

The complete reading of the texts and extraction of data from the original articles were independently conducted by two authors (J.V.S.R. and M.B.D.). The electronic search was performed up to July 2024 (subsequently updated by alerts) in the PubMed/MEDLINE, Scopus, and Web of Science libraries. There were no restrictions on publication data and languages. Open Grey (opengrey.eu), a gray literature database, was utilized to identify eligible studies in the gray literature. Following a thorough examination of the full articles, the primary data were extracted, tabulated, and cross-checked between both researchers. Any discrepancies that arose were resolved through meetings and collaborative discussions, guided by predefined criteria. In instances where doubts or conflicts in data extraction occurred, a third author (R.S.d.M.) was consulted. 

The tabulation of the studies encompassed several key parameters, including the authors and the country of origin of the research, study design, method of antibiotic therapy prescription, therapies under investigation, number of patients per group (n), treatment duration, and main findings of each study. Additionally, the tabulation included parameters related to proposed clinical treatments and numerical data presenting mean values and standard deviations for the difference in means between baseline and the analyzed periods for PPD and CAL.

### 2.6. Risk of Bias

Bias risk was evaluated using the Cochrane Collaboration tool [31]. For each domain, bias risk was judged according to the following classification: (1) low risk of bias (when all criteria were properly met); (2) unclear risk (when there was insufficient information to make a judgment); (3) high risk of bias (when two or more criteria were not met). All these domains were applied in each study. Two reviewers (J.V.S.R. and M.B.D.) independently conducted the quality assessment, with any disagreements resolved through consultation with a third investigator (R.S.d.M.).

The studies were assessed and classified across the following domains:

1. Random sequence generation;
2. Allocation concealment;
3. Blinding of participants and personnel;
4. Blinding of outcome assessment (patient-reported outcomes);
5. Blinding of outcome assessment (all-cause mortality);
6. Incomplete outcomes (short-term [2–6 weeks]);
7. Incomplete outcomes (long-term [>6 weeks]);
8. Selective outcome reporting.

Each domain was deemed as adequate (+), inadequate (−), or unclear (?). Bias analysis was presented and categorized based on the association between topical and systemic antibiotics.

### 2.7. Data Synthesis and Meta-Analysis

The synthesis of qualitative results followed the PRISMA 2020 Statement [33]. Descriptive results were presented in the form of text, figure, and tables, including study selection, study characteristics, risk of bias within studies, results of individual studies, results of syntheses, reporting biases, and certainty of evidence.

Meta-analysis of effect estimates consisted of standard pairwise meta-analyses of direct comparisons (inverse variance meta-analytical method) using a random-effect model. The results were expressed as standardized mean difference and relative 95% CI. Heterogeneity was assessed by Q-statistic method (*p* ≤ 0.1) and inconsistency measurement (I^2^ ≥ 75% suggesting high heterogeneity) [34,35]. Meta-analyses with *p* ≤ 0.1 in the χ^2^ and I^2^ ≥ 75% were not included in the data synthesis. Statistical tests were performed using RevMan 5.4 software (Review Manager (RevMan) [Computer program], Version 5.4, The Cochrane Collaboration, 2020).

### 2.8. Certainty of Evidence

Certainty of evidence was evaluated following the GRADE approach, adapting all the judgments to qualify the evidence in a narrative way [36,37]. The risk of bias, inconsistency, indirectness, imprecision, and other information (suspicion of publication bias, presence of large effect, dose–response gradient, and plausible confounders) were the items considered to rate the overall certainty of evidence. Additional parameters such as effect size, dose–response gradient, and residual confounding factors could increase the quality of the evidence. Thus, the evidence quality index was defined in four categories, namely high, moderate, low, and very low, which were applied to each of the evaluated outcomes [38,39,40]. 

## 3. Results

### 3.1. Selection of Studies

**Figure 1:**
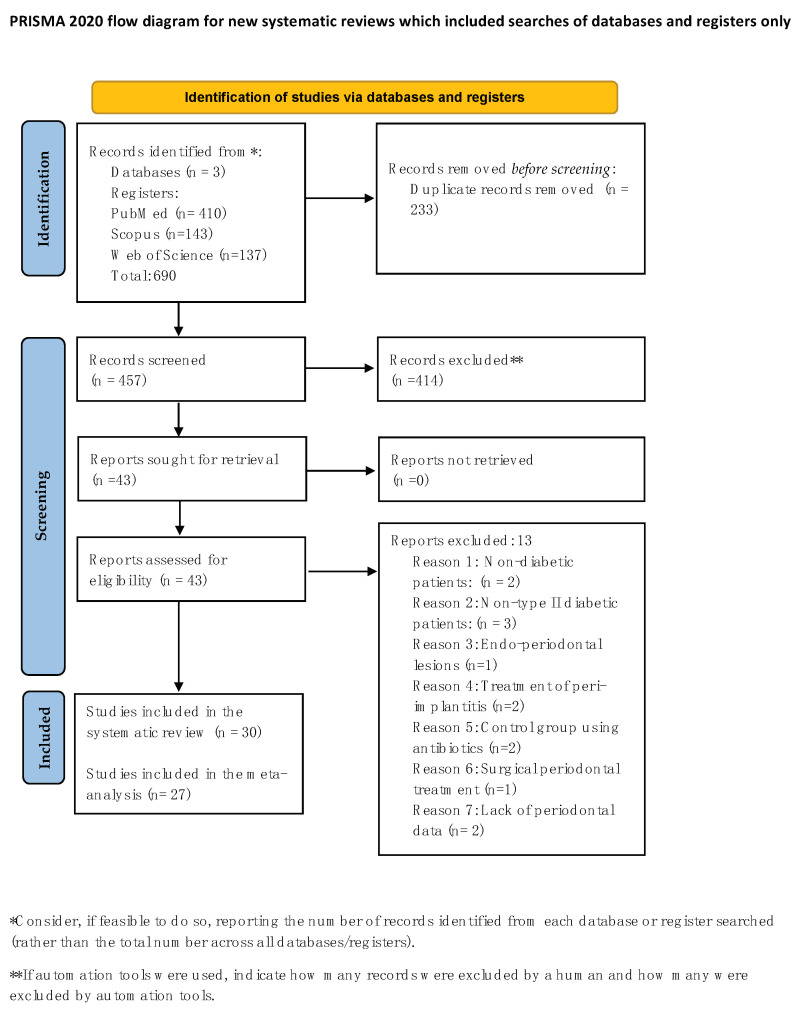
PRISMA flowchart of the included studies from [33].

A total of 690 studies were initially identified across the MEDLINE/PubMed, Scopus, and Web of Science platforms. Following the removal of duplicates (233 articles), 457 records screened remained. Upon screening titles, 414 articles were excluded for failing to meet the eligibility criteria. Subsequently, 43 articles underwent abstract screening, leading to the selection of articles for full-text review. Among these, thirteen were excluded due to the following reasons, as described in Figure 1: two studies utilized non-diabetic patients; three studies involved type 1 diabetes mellitus; one study presented patients with endo-perio lesions; two studies had no control group; one study involved periodontal surgery; two studies were related to peri-implantitis; and two did not provide periodontal data. Consequently, 30 studies were included for data extraction and qualitative analysis. Figure 1 illustrates the study identification flowchart according to the PRISMA guidelines, delineating the reasons for exclusion of abstracts and full texts.

### 3.2. General Characteristics of the Included Studies

Thirty randomized clinical trials were included in this systematic review. Nine studies focused on the topical administration of antibiotics. Of these, three were conducted in India [41,42,43], one in Taiwan [44], three in Japan [45,46,47], one in Poland [48], and one in Brazil [49]. These studies assessed the topical use of antibiotics at various dosages (as described in Table 1) as adjuvants in NSPT. The included studies utilized a varied of antibiotics, i.e., minocycline, doxycycline, clarithromycin, azithromycin, and tetracycline.

The remaining twenty-one studies investigated the systemic administration of antibiotics. These were conducted in Saudi Arabia [50,51,52], Colombia [53,54,55], India [26,56], Thailand [57], Greece [58], China [59], France [28], Brazil [60,61,62,63,64], Egypt [27,65], Japan [66], and Pakistan [67]. These studies evaluated the use of systemic antibiotics at different dosages (as described in Table 2) as adjuvants in NSPT, covering a variety of antibiotics, such as doxycycline, azithromycin, amoxicillin, and metronidazole.

### 3.3. Description and Results of Studies with the Combination of Topical Antibiotics 

Table 1 lists the nine studies on the topical administration of antibiotics in individuals with DM2 and periodontitis. In all studies, both groups received SRP with or without a placebo, except for the study by Katagiri et al. [46], where the control group received only oral hygiene instructions. In this study, the test group, in addition to SRP, received the drug as adjunct therapy. The dosages and frequency of drug administration varied among the studies. The re-evaluation periods also varied, ranging from 1 month [42,43,46,49], 2 months [45], 3 months [41,42,43,44,46,48,49], 4 months [47], and 6 months [41,42,43,44,45,46,49] to 9 months [41,47].

The majority of the studies using the topical administration of antibiotics utilized minocycline [44,45,46,47] at different dosages varying from 10 mg [45,46,47] to 2% gel [44,47]. Subantimicrobial doses (20 mg) of doxycycline were used by Gilowski et al. [48]. Additionally, 20% doxycycline-loaded PLGA nanospheres were used as adjunctive therapy to NSPT [49]. Other antibiotics included clarithromycin gel at 0.5% [42], azithromycin at 0.5% [41], and satranidazole gel at 3% [43]. The majority of the included studies applied the antibiotics into the periodontal pocket with the aid of a syringe. The type of study, number of patients included, the intervention and dosages utilized, study duration, and the main results obtained by the included studies are described in Table 1.

### 3.4. Description and Results of Studies with the Combination of Systemic Antibiotics 

Table 2 lists the twenty-one studies on the systemic administration of antibiotics in individuals with DM2 and periodontitis. In all studies, both groups received SRP treatment with or without a placebo, while the test group, in addition to SRP, had the drug association as adjunct therapy. However, the dosages and frequency of drug administration varied among the studies. The period of re-evaluations varied in the studies, i.e., 1 month [50,52,56,65,66,67], 2 months [56] 3 months [26,27,50,51,52,53,54,55,56,57,60,61,62,66,67], 4 months [56], 6 months [50,53,55,58,60,66,67], 9 months [53,55,66], 1 year [60,63], 2 years [63,64], and 5 years [64].

The majority of the studies evaluated the effects of systemic antibiotic administration using doxycycline [26,50,51,52,54,56,57,58,61], utilizing different concentrations, such as 20 mg [52], 100 mg [26,50,51,54,56,57,58,61], and 200 mg [58,61]; others used 500 mg and 2000 mg azithromycin [53,55,66], metronidazole [27,59,60,63,64,65,67] in different concentrations, varying from 200 mg [59] and 400 mg [27,60,63,64,67] to 500 mg [65], 500 mg [27,59,60,63,64,65,68] or 2 g [28] amoxicillin, and 875 mg amoxicillin/clavulanic acid [62]. The type of study, number of patients included, the intervention and dosages utilized, the study duration, and the main results obtained by the included studies are described in Table 2.

### 3.5. Bias Analysis

**Figure 2:**
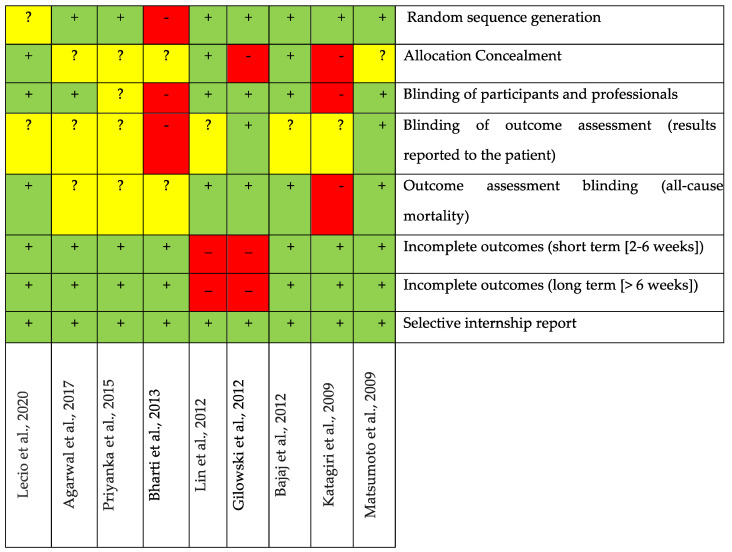
Evaluation of risk of bias of the included randomized clinical trials on the topical application of antibiotics. Green + color means low risk of bias (when all criteria were properly met); yellow? color means unclear risk (when there was insufficient information to make a judgment); red−color means high risk of bias (when two or more criteria were not met) [41,42,43,44,45,46,47,48,49].

The risk of bias assessment of the selected studies utilizing topical applications of antibiotics in randomized clinical trials is presented in Figure 2. In summary, five studies [41,42,43,47,49] were classified as having a low risk of bias. Conversely, four studies [44,45,46,48] were identified as having a high risk of bias, i.e., did not meet two or more criteria. Seven studies reported random sequence generation and only two studies reported allocation concealment. In six studies [41,42,44,47,48,49], the authors described the blinding of participants and professionals; however, the blinding of outcome assessment was reported only by two studies [47,48]. Therefore, six studies were classified as having unclear risk for this parameter (as there was insufficient information to make a judgment). Only two studies [44,48] did not describe incomplete outcomes and were considered to have a high risk of bias. Five studies [41,42,43,47,49] were apparently free of other problems that could result in a high risk of bias.

**Figure 3:**
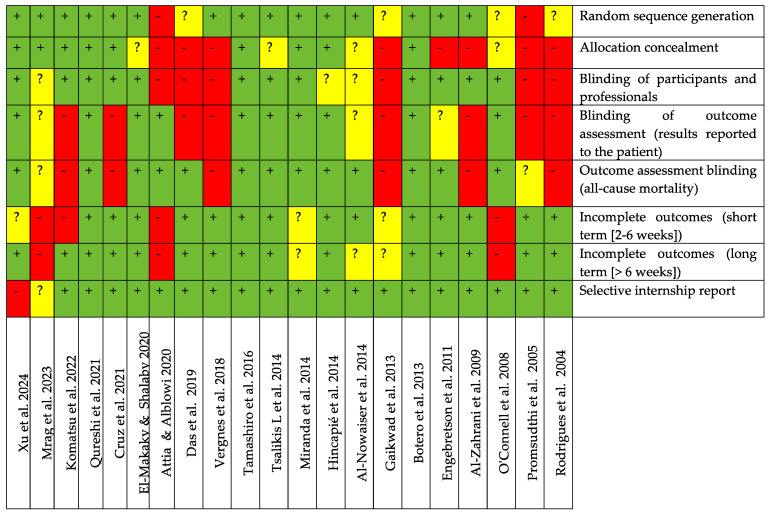
Evaluation of risk of bias of the included randomized clinical trials on the systemic application of antibiotics. Green + color means low risk of bias (when all criteria were properly met); yellow? color means unclear risk (when there was insufficient information to make a judgment); red−color means high risk of bias (when two or more criteria were not met) [26,27,28,50,51,52,53,54,55,56,57,58,59,60,61,62,63,64,65,66,67].

The risk of bias assessment of the selected studies utilizing the systemic administration of antibiotics in randomized clinical trials is presented in Figure 3. In summary, of the twenty-one included studies [26,27,50,51,52,53,54,55,56,60,61,64,65,66], eight were classified as having a low risk of bias [27,50,53,55,58,60,63,67]; two studies were classified as having an undefined risk [54,59], because one parameter was defined as obscure; and eleven studies showed a high risk of bias [26,28,51,52,56,57,61,62,64,65,66], with two or more parameters classified as high-risk. Fifteen studies reported random sequence generation [27,50,51,53,54,55,58,60,63,64,65,66,67], but allocation concealment was described in only nine studies [53,55,59,60,63,64,65,66,67]. The blinding of participants and professionals was described in twelve studies [27,51,53,54,58,59,60,61,63,64,66,67], and ten studies reported the blinding of outcome assessment [27,52,53,55,58,59,60,61,63,67]. Of the eleven studies classified to have a high risk of bias, eight did not report the blinding of outcome assessment [26,28,51,56,61,62,64,66], eight did not report allocation concealment [26,28,51,52,54,56,61,62], six studies did not describe the blinding of participants and professionals [26,28,52,56,61,62], and six did not report outcome assessment blinding [28,51,56,62,64,66]. Therefore, all of these eleven studies [26,28,51,52,56,57,61,62,64,65,66] were judged as having a high risk of bias.

### 3.6. Main Results and Meta-Analysis

In extracting individual results from each study according to the period of evaluation and also regarding the individual analysis of each type of antibiotics utilized in the NSPT of diabetic patients as an adjunct to SRP, most authors reported no significant benefits of using antibiotics for clinical periodontal parameters, such as PPD reduction, CAL gain, and BoP reduction, when topical or systemic antibiotics were used compared to placebo. 

**Figure 4:**
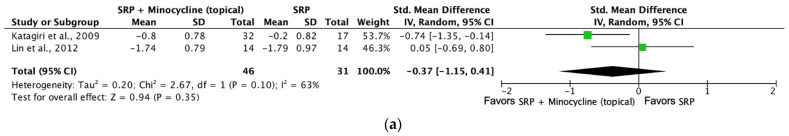

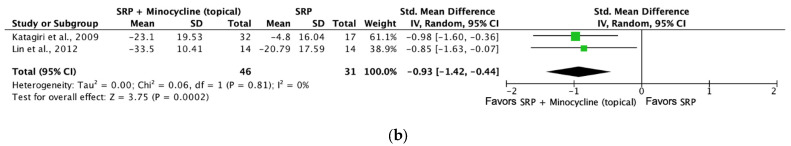
Forest plot comparing SRP + minocycline versus SRP in non-surgical periodontal treatment [44,46]. (**a**) Forest plot comparing SRP + minocycline versus SRP in non-surgical periodontal treatment, PPD reduction (mm). (**b**) Forest plot comparing SRP + minocycline versus SRP in non-surgical periodontal treatment, BoP (%). SRP, scaling and root planing; PPD, probing pocket depth; BoP, bleeding on probing.

For the meta-analysis of the topical application of minocycline at three months’ follow-up period, the clinical periodontal parameter PPD (Figure 4a) was not statistically significant compared to the placebo group. On the other hand, BoP (Figure 4b) demonstrated significant improvements in favor of antibiotics plus SRP.

**Figure 5:**
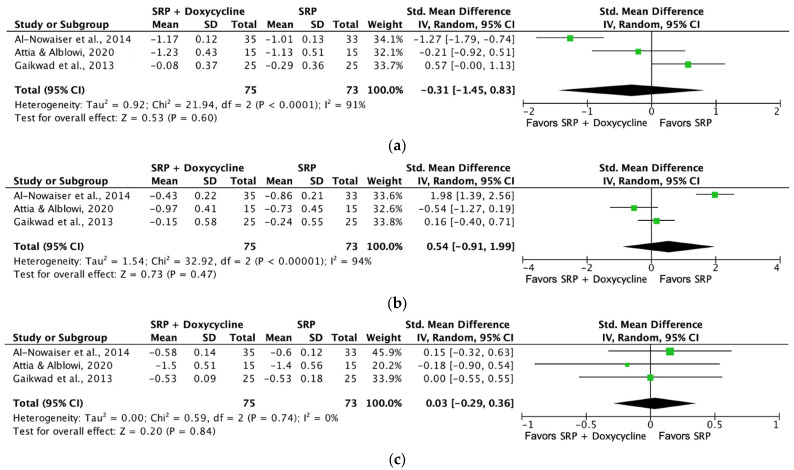
Forest plot comparing SRP + doxycycline versus SRP in non-surgical periodontal treatment at one month of follow-up [50,52,56]. (**a**) Forest plot comparing SRP + doxycycline versus SRP in non-surgical periodontal treatment, PPD reduction (mm). (**b**) Forest plot comparing SRP + doxycycline versus SRP in non-surgical periodontal treatment, CAL gain (mm). (**c**) Forest plot comparing SRP + doxycycline versus SRP in non-surgical periodontal treatment, PI (%). SRP, scaling and root planing; PPD, probing pocket depth; CAL, clinical attachment level (%), PI, plaque index (%).

For the meta-analysis of the systemic application of doxycycline at one month’s follow-up, the clinical periodontal parameters PPD (Figure 5a), CAL (Figure 5b), and PI (Figure 5c) were not statistically significant compared to the placebo groups.

**Figure 6:**
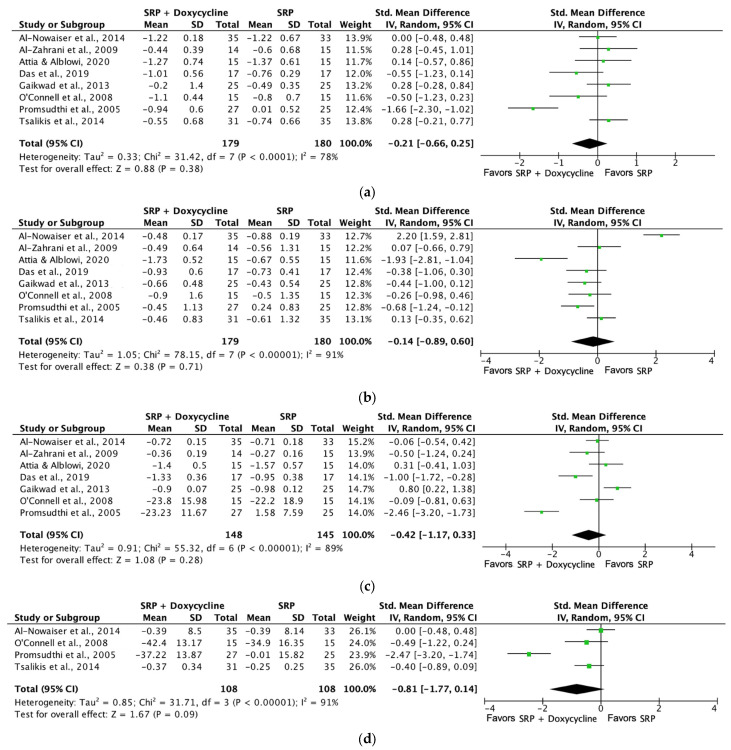
Forest plot comparing SRP + doxycycline versus SRP in non-surgical periodontal treatment after 3 months of follow-up [26,50,51,52,56,57,58,61]. (**a**) Forest plot comparing SRP + doxycycline versus SRP in non-surgical periodontal treatment, PPD reduction (mm). (**b**) Forest plot comparing SRP + doxycycline versus SRP in non-surgical periodontal treatment, CAL gain (mm). (**c**) Forest plot comparing SRP + doxycycline versus SRP in non-surgical periodontal treatment, PI (%). (**d**) Forest plot comparing SRP + doxycycline versus SRP in non-surgical periodontal treatment, BoP (%). SRP, scaling and root planing; PPD, probing pocket depth; CAL, clinical attachment level (%), PI, plaque index (%), BoP, bleeding on probing.

Figure 6 shows the forest plot for (a) PPD reduction, (b) CAL gain, (c) PI, and (d) BoP analyzing the mean differences in PPD (mm), CAL gain (mm), PI (%), and BoP (%) at three months in control groups with SRP and test groups using SRP + systemic doxycycline therapy. All the evaluated parameters were not significant when systemic antibiotics were associated with SRP for the parameters evaluated.

**Figure 7:**
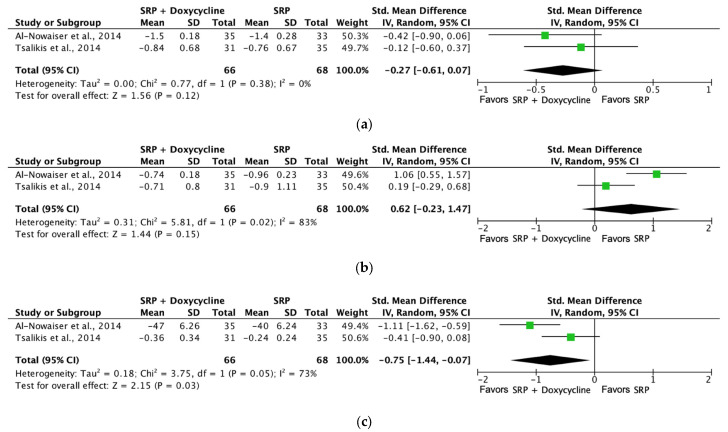
Forest plot comparing SRP + doxycycline versus SRP in non-surgical periodontal treatment after 6 months of follow-up [50,58]. (**a**) Forest plot comparing SRP + doxycycline versus SRP in non-surgical periodontal treatment, PPD reduction (mm). (**b**) Forest plot comparing SRP + doxycycline versus SRP in non-surgical periodontal treatment, CAL gain (mm). (**c**) Forest plot comparing SRP + doxycycline versus SRP in non-surgical periodontal treatment, BoP (%). SRP, scaling and root planing; PPD, probing pocket depth; CAL, clinical attachment level (%); BoP, bleeding on probing.

Figure 7 shows the forest plot for (a) PPD reduction, (b) CAL gain, and (c) BoP analyzing the mean differences in PPD (mm), CAL gain (mm), and BoP (%) at six months in control groups with SRP and test groups using SRP + systemic doxycycline therapy. PPD and CAL gain were not significantly different when systemic antibiotics were associated with SRP. However, there were significant improvements in the percentage of BoP favoring the use of systemic antibiotics after 6 months of treatment. However, only two studies were included, and the interpretation of these findings should be carefully considered.

**Figure 8:**
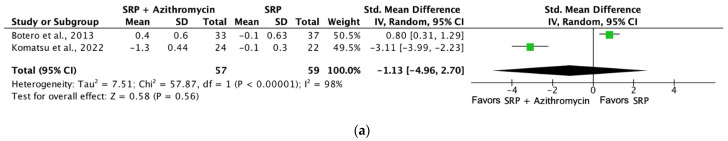

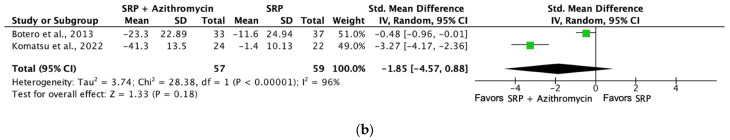
Forest plot comparing SRP + azithromycin versus SRP in non-surgical periodontal treatment after 3 months of follow-up [53,66]. (**a**) Forest plot comparing SRP + azithromycin versus SRP in non-surgical periodontal treatment, PPD reduction (mm). (**b**) Forest plot comparing SRP + azithromycin versus SRP in non-surgical periodontal treatment, CAL gain (mm). SRP, scaling and root planing; PPD, probing pocket depth; CAL, clinical attachment level.

Figure 8 shows the forest plot for (a) PPD reduction and (b) CAL gain analyzing the mean differences in PPD (mm) and CAL gain (mm), at three months with SRP versus SRP + systemic azithromycin therapy. PPD and CAL gain were not significantly different when systemic azithromycin was associated with SRP. 

**Figure 9:**
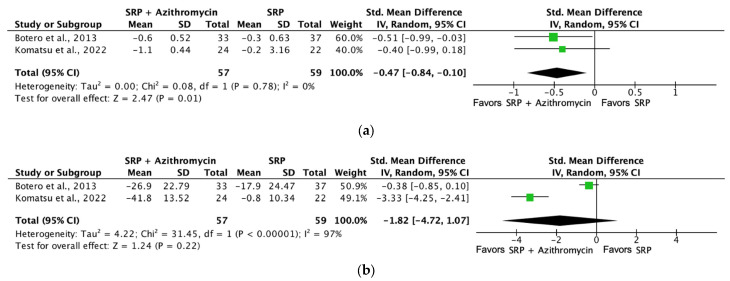
Forest plot comparing SRP + azithromycin versus SRP in non-surgical periodontal treatment after 6 months of follow-up [53,66]. (**a**) Forest plot comparing SRP + azithromycin versus SRP in non-surgical periodontal treatment, PPD reduction (mm). (**b**) Forest plot comparing SRP + azithromycin versus SRP in non-surgical periodontal treatment, BoP (%). SRP, scaling and root planing; PPD, probing pocket depth; BoP, bleeding on probing.

Figure 9 shows the forest plot for (a) PPD reduction and (b) BoP analyzing the mean differences in PPD (mm) and BoP (%), at six months with SRP versus SRP + systemic azithromycin therapy. Improvements in PPD were statistically significant when compared to the control group. On the other hand, BoP was not significantly different when systemic azithromycin was associated with SRP.

**Figure 10:**
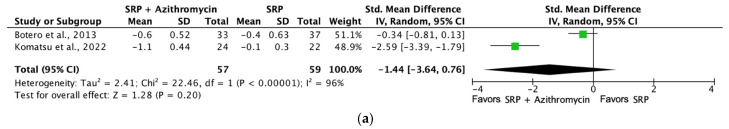

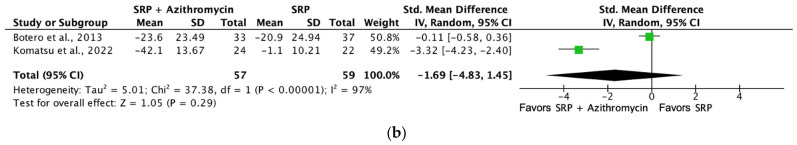
Forest plot comparing SRP + azithromycin versus SRP in non-surgical periodontal treatment after 9 months of follow-up [53,66]. (**a**) Forest plot comparing SRP + azithromycin versus SRP in non-surgical periodontal treatment, PPD reduction (mm). (**b**) Forest plot comparing SRP + azithromycin versus SRP in non-surgical periodontal treatment, BoP (%). SRP, scaling and root planing; PPD, probing pocket depth; BoP, bleeding on probing.

Figure 10 shows the forest plot for (a) PPD reduction and (b) BoP analyzing the mean differences in PPD (mm) and BoP (%), at nine months with SRP versus SRP + systemic azithromycin therapy. Neither PPD levels nor the percentage of BoP demonstrated any significant difference when systemic azithromycin was associated with SRP.

**Figure 11:**
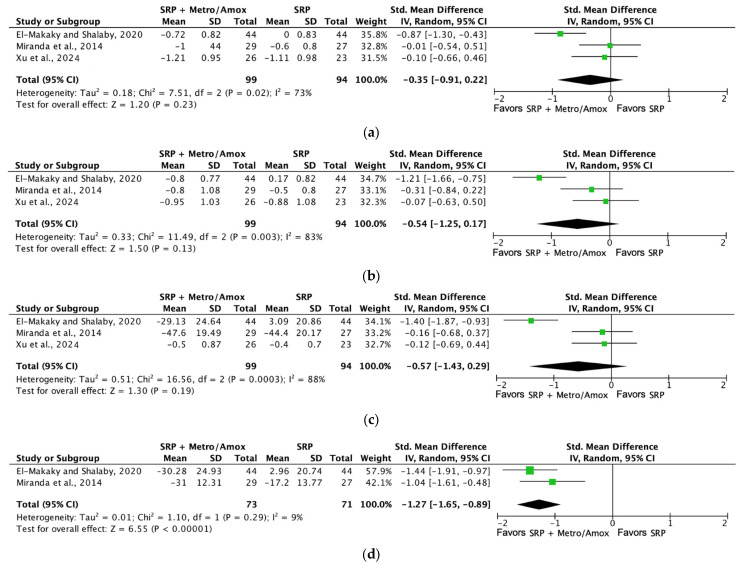
Forest plot comparing SRP + metronidazole/amoxicillin versus SRP in non-surgical periodontal treatment after 3 months of follow-up [27,59,60]. (**a**) Forest plot comparing SRP + metronidazole/amoxicillin versus SRP in non-surgical periodontal treatment, PPD reduction (mm). (**b**) Forest plot comparing SRP + metronidazole/amoxicillin versus SRP in non-surgical periodontal treatment, CAL gain (mm). (**c**) Forest plot comparing SRP + metronidazole/amoxicillin versus SRP in non-surgical periodontal treatment, PI (%). (**d**) Forest plot comparing SRP + metronidazole/amoxicillin versus SRP in non-surgical periodontal treatment, BoP (%). SRP, scaling and root planing; PPD, probing pocket depth; CAL, clinical attachment level (%), PI, plaque index (%), BoP, bleeding on probing.

Figure 11 shows the forest plot for (a) PPD reduction, (b) CAL gain, (c) PI, and (d) BoP analyzing the mean differences in PPD (mm), CAL gain (mm), PI (%), and BoP (%) at three months with SRP versus SRP + systemic metronidazole/amoxicillin therapy. Only BoP improved after treatment with systemic antibiotics.

### 3.7. Certainty of Evidence

The effects of systemic antibiotics as adjuvants to NSPT on the clinical parameters evaluated in this meta-analysis were classified as having a high certainty of evidence for PD (SRP + doxycycline [6 months] and SRP + Metro/Amox [3 months]) and BoP (SRP + doxycycline [6 months] and SRP + Metro/Amox [3 months]) and a moderate certainty of evidence for PI (SRP + doxycycline [1 month]), PD (SRP + azithromycin [6 months] and SRP + minocycline—topical application route [3 months]), and BoP (SRP + minocycline—topical application route [3 months]). For more details on the GRADE assessment, see Table 3.

## 4. Discussion

Individuals with DM exhibit a range of metabolic alterations, primarily affecting the immune system’s response to bacterial aggression and circulating inflammatory mediators, including pro-inflammatory cytokines, chemokines, and prostaglandins, which are known to be exacerbated in these individuals [69]. It is noteworthy that in patients with DM, connective tissue metabolism is compromised due to a reduced function and number of fibroblasts, leading to decreased collagen levels and a heightened susceptibility to connective tissue destruction [70]. Severe forms of periodontitis are known to negatively impact the outcomes of glycemic control of DM patients. Therefore, studies investigating the benefits of antimicrobials to manage periodontitis in DM patients are ones that should be explored more to come to more definitive conclusions about their beneficial effects in this class of affected patients.

Therefore, our goal in this systematic review was to evaluate the hypothesis that topical or systemic antibiotic therapy, as an adjunct to SRP, would yield superior clinical outcomes compared to SRP alone. Additionally, it aimed to assess the actual effectiveness of antibiotic use as adjunctive therapy for controlling periodontitis in patients with DM, thus fostering new perspectives for their recommendation while curbing their indiscriminate use, which could exacerbate bacterial resistance to antibiotics.

Early diagnosis typically allows for clinical improvements through NSPT, manifesting in reduced PPD, BoP, and, in some instances, a gain in CAL. However, the direct association with DM significantly impacts the progression of periodontitis due to metabolic and immunological alterations in affected individuals. NSPT is the preferred treatment for periodontitis stages 1 to 3, although adjunctive treatments, such as antibiotic therapy, are often indicated in certain cases [71,72]. This review highlights a diversity of treatment plans, dosages, procedures, and antibiotic selections, signifying ongoing efforts to enhance therapeutic options with favorable outcomes, notwithstanding the existing gaps in the literature.

The choice of antibiotic administration method necessitates the consideration of several factors. Local or topical antibiotics offer advantages such as achieving high concentrations at specific sites, treatment adherence without the need for continuous patient compliance, minimal adverse effects, and reduced systemic risk of drug resistance development. However, they also present limitations, including challenges in precise drug delivery to the site, requiring skilled application, and the inability to reach adjacent periodontal areas, thereby increasing the risk of reinfection or recurrence of treated diseases.

In studies evaluating adjunctive systemic antibiotic therapy, significant variations were observed in terms of administration period, dosage, number of participants, treatment peculiarities, and antibiotic class. The use of systemic antimicrobials can pose risks such as promoting bacterial resistance. Therefore, the decision to utilize these medications should be based on a thorough analysis of the patient’s periodontal and systemic clinical condition, the drug’s spectrum of action, and its mechanism of action to avoid unreasonable use.

Doxycycline emerged as the most discussed antibiotic in systemic studies, examined by nine studies [26,50,51,52,54,56,57,58,61]. Doxycycline is favored for its affordability, relatively good tolerance, broad spectrum of action, and ability to achieve higher concentrations in periodontal pockets when administered systemically [73]. It also benefits diabetic patients by inhibiting metalloproteinases that contribute to collagen fiber breakdown in periodontal tissues. Moreover, its complete absorption by the intestine interferes less with the native gastrointestinal microbiota, potentially justifying its frequent selection in the systemic studies reviewed in this systematic review.

In adjunctive treatment with amoxicillin without combination with another medication, as studied by Vergnes et al. [28], positive results were observed at the periodontal level compared to the test group, although no significant effects on glycemic control were noted. Similarly, the association of amoxicillin with clavulanic acid, as investigated by Rodrigues et al. [62], demonstrated improvements in the analyzed parameters, albeit without statistical significance compared to SRP alone.

A recent guideline by the European Federation of Periodontology suggested systemic antibiotic therapy should be considered only in specific cases, such as in young adult patients with generalized stage 3 periodontitis [24]. While this meta-analysis suggests favorable outcomes with systemic antibiotic therapy as an adjunct to SRP in treating periodontitis at 6 months, it should not be the primary treatment choice. Emphasis should be placed on patient follow-up with maintenance therapies, motivation for plaque control, and regular oral hygiene reinforcement, alongside initial NSPT with SRP. Early diagnosis enables clinical improvements, particularly in reducing CAL and, in some instances, periodontal pocket depth [74].

Although no statistically significant differences were observed in CAL gain with systemic antibiotics adjunctive to SRP compared to SRP alone, this meta-analysis revealed advantages in PPD reduction with azithromycin, underscoring the importance of the judicious clinical use of antimicrobials and tailored dosages for each patient. Indeed, our findings suggest that antibiotics adjuvant to NSPT offer some clinical benefits. Comparing our results with previously published systematic reviews, it was observed that when all antibiotic protocols for the adjunctive treatment of patients with periodontitis were considered together, a significant, albeit small, reduction in PPD was observed, with no improvement in CAL gain [75]. However, when these antibiotics are considered separately, it is clear that the combination of amoxicillin and metronidazole showed the best results in reducing PPD [75]. Systemic amoxicillin–metronidazole may yield better clinical outcomes when combined with NSPT [76], offering adjuvant clinical benefits of antimicrobial therapy in terms of PPD reduction [77,78] and CAL gain, especially in well-controlled individuals and in deep periodontal pockets [78]. However, doxycycline did not show a significant improvement in clinical attachment levels [79], which parallels the observations made in our study.

Regarding topical antibiotics, diverse application regimens and antibiotic classes were evaluated in this review, all showing subtle improvements in predetermined clinical parameters such as PPD, CAL, GI, and BoP. Four studies focused on minocycline, a semi-synthetic derivative of tetracyclines, known for its broad antibacterial activity [44,45,46,47]. These biodegradable polymers adhere to the periodontal pocket wall, maintaining bacteriostatic concentrations in crevicular fluid for up to 14 days, thereby inhibiting pathogenic bacteria and facilitating clinical improvement.

Results from studies on minocycline demonstrated some favorable clinical outcomes compared to baseline. Despite being a topical treatment, its effects had systemic implications, as observed in the study by Skaleric et al. [80], suggesting a bidirectional relationship between DM and periodontitis. While SRP alone proved effective, the addition of minocycline conferred additional benefits in some cases, especially regarding BoP, with the exception of one study by Lin et al. [44], which reported no significant differences between the test and control groups despite improvements in both. The results from the meta-analyses demonstrated only a reduction in BoP when topical antibiotics were applied in comparison with only SRP.

The heterogeneity index, represented by I2, indicated variations in clinical parameters among studies evaluating topical or systemic antibiotics as adjuncts to SRP. These variations may be attributed to differences in SRP techniques, operator proficiency, probing force, patient demographics, and DM diagnosis criteria. Therefore, as the majority of the included studies presented with a high heterogeneity of data, the results of this systematic review and meta-analyses should be carefully considered when evaluating the achieved outcomes.

Despite the subtle benefits of antibiotic therapy as an adjunct to NSPT in diabetic patients, their use should be based on a thorough diagnosis of the patient’s clinical condition and systemic health, considering the rising prevalence of multidrug-resistant bacteria associated with indiscriminate antibiotic use [81,82]. Therefore, antibiotic therapy for dental procedures, including periodontal treatment, should adhere to specific guidelines and be tailored to individual patient characteristics.

It is important to note that our study has some limitations. Firstly, there are a low number of studies included that compare each evaluated outcome (clinical periodontal parameters) across different periods of analysis. Similarly, the meta-analysis results include few studies, particularly regarding the topical application of antibiotics. Therefore, these results should be interpreted with caution. Based on the findings presented in this review, it is reasonable to consider that the adjunctive use of antibiotics (topical or systemic) associated with SRP shows subtle improvements in the evaluated periodontal parameters. Due to the increase in microbial resistance within the global population, the systemic use of antibiotics should be prescribed judiciously.

The influence of topical or systemic antibiotics in addition to NSPT on diabetic patients is a relevant and evolving field of study. However, several future directions can be explored to enhance the adjunctive treatment of periodontitis through antibiotics in diabetic patients. These include conducting long-term longitudinal studies to investigate the lasting effects of topical and systemic antibiotics on periodontal health, exploring new antibiotics and combinations, examining the impact of antimicrobial resistance on the efficacy of antibiotics, and considering side effects and safety. Additionally, alternative and complementary approaches, as well as education and awareness, should be addressed. These directions can help improve the effectiveness of NSPT in diabetic patients by providing a deeper understanding of the interactions between antibiotics, diabetes, and periodontal health.

## 5. Conclusions

In conclusion, incorporating topical or systemic antibiotic therapy into the treatment of periodontitis in diabetic patients offers subtle clinical benefits (especially regarding inflammation reduction, represented by a decreased BoP percentage). However, the potential risks associated with antibiotic use, such as the emergence of resistant bacteria and side effects, necessitate judicious prescription practices. Given the lack of consensus regarding optimal administration timing and long-term benefits, an individualized approach based on the patient’s evolving clinical condition is recommended.

## Figures and Tables

**Table 1:** List and description of clinical studies involving topical antibiotics.

Study Country	Type of Study	Number of Patients	Interventions	Study Duration	Results
Matsumoto et al. (2009) [47] Japan	Randomized clinical trial	21 individuals with DM2: - Test: 11 patients - Control: 10 patients	- Test: SRP + OHI and minocycline gel at 2% applied locally in each periodontal pocket - Control: SRP + mechanic cleaning of teeth	9 months	Clinical parameters of the test group turned out to be better. The % of sites with PPD ≥ 4 mm showed a reduction of 8.3% and 9.3% after the administration of the gel, compared to just 5% in the control group.
Katagiri et al. (2009) [46] Japan	Parallel to control	49 individuals with DM2: - Test: 32 patients - Control: 17 patients	- Test: SRP + 10 mg minocycline applied in all the periodontal pockets - Control: OHI	6 months	PPD and BoP improved in the 1st month compared to the baseline, and these improvements were maintained during the study period for the test group. In the control group, the parameters were somewhat reduced at 6 months compared to the beginning of treatment.
Lin et al. (2012) [44] Taiwan	Randomized controlled clinical trial	28 individuals with DM2: - Test: 14 patients - Control: 14 patients	- Test: SRP + 2% minocycline gel - Control: SRP	6 months	Both groups showed improvements in the periodontal levels of PPD, BoP, and CAL, as well as benefits in DM2 control, but without significant differences between them.
Bajaj et al. (2012) [42] India	Randomized controlled clinical trial	56 individuals with DM2: - Test: 29 patients - Control: 27 patients	- Test: SRP + 0.5% clarithromycin gel - Control: SRP + gel - placebo	6 months	Both therapies resulted in significant clinical improvements. Test group demonstrated improved reductions in PI, GI, SBI, and PPD and gains in CAL after 6 months as compared to control.
Gilowski et al. (2012) [48] Poland	Randomized study	34 individuals with DM2: - Test: 17 patients - Control: 17 patients	- Test: SRP + 20 mg of doxycycline hydrochloride twice a day - Control: SRP + placebo twice a day	3 months	CAL, PPD, and BOP improved significantly in both groups after therapy. Difference between the two groups after therapy was seen in PPD in tooth sites with initial PPD ≥ 4 mm (SI + placebo: 3.41 ± 0.6 mm vs SI + doxycycline: 2.92 ± 0.5 mm, *p* < 0.05)
Bharti et al. (2013) [45] Japan		29 individuals with DM2: - Test: 21 patients - Control group: 8 patients	- Test: SRP + 10 mg minocycline 4 times every two weeks - Control: no periodontal treatment	6 months	The test group demonstrated improvements in PPD and BoP starting at 2 months and continuing for 6 months. In the control group, PPD and BoP were not changed. BoP was significantly lower in the test group compared to the control group at baseline and after 6 months.
Priyanka et al. (2015) [43] India	Randomized controlled clinical trial	57 individuals with DM2: - Test: 28 patients - Control: 29 patients	- Test: SRP + 3% satranidazole - Control: SRP + local delivery of a placebo gel	6 months	PI and GI were reduced, albeit without significant difference between the two groups. GI and PPD were significantly reduced at 3 and 6 months compared to baseline. CAL gain was significantly greater in the test group compared to the control group at all periods.
Agarwal et al. (2017) [41] India	Randomized controlled clinical trial	56 individuals with DM2: - Test: 27 patients - Control: 29 patients	- Test: SRP + 0.2 mL azithromycin gel a 0.5%. - Control: SRP + placebo	9 months	Both therapies resulted in significant clinical improvements. Patients in the test group showed enhanced reductions in PI, GI, SBI, and PPD and gains in CAL over 9 months compared with the control group.
Lecio et al. (2020) [49] Brazil	Parallel, double-blind, placebo-controlled clinical trial	40 individuals with DM2: - Test: 20 patients - Control: 20 patients	- Test: SRP + 20% doxycycline in nanospheres - Control: SRP + nanospheres in placebo	6 months	Both groups showed clinical improvement in all parameters after treatment (*p* < 0.05). Deep pockets showed improvements in BoP (3 and 6 months), PPD (at 3 months), and CAL gain (at 1 and 3 months). The percentage of sites presenting PPD reduction and CAL gain ≥ 2 mm was higher in the test group at 3 months.

Abbreviations: DM2—diabetes mellitus type 2; SRP—scaling and root planing; USA—United States; OHI—oral hygiene instructions; PPD—probing pocket depth; BoP—bleeding on probing; PS—plaque score; CAL—clinical attachment level; PI—plaque index; GI—gingival index; SBI—sulcus bleeding index.

**Table 2:** List and description of clinical studies involving systemic antibiotics.

Study Country	Type of Study	Number of Patients	Interventions	Study Duration	Results
Rodrigues et al. (2004) [62] Brazil	Parallel, double blind, control	30 individuals with DM2: - Test: 15 patients - Control: 15 patients	- Test: SRP + 875 mg amoxicillin and clavulanic acid twice a day for 14 days - Control: SRP	3 months	Both groups presented clinical periodontal improvements. There was a reduction in PPD of 0.8 ± 0.6 mm in the test and 0.9 ± 0.4 mm in the control group. No significant changes in CAL were observed. At the 3-month mark, an improvement in PPD was recorded for both treatment groups. The test group showed a mean PPD reduction from 2.7 ± 0.7 mm to 1.9 ± 0.4 mm and the control group from 3.2 ± 0.8 mm to 2.3 ± 0.5 mm. No changes in CAL levels were recorded. Both groups presented an improvement in BoP. The test group showed a reduction from 38 ± 13% to 15 ± 9% and the control group from 32 ± 15% to 11 ± 7%.
Promsudth i et al. (2005) [57] Thailand	Parallel, double blind, placebo control	52 individuals with DM2: - Test: 27 patients - Control: 25 patients	- Test: OHI + SRP + 100 mg doxycycline once daily for 14 days - Control: SRP + OHI and no antibiotic treatment	3 months	PPD and CAL were not different between the groups. However, the mean PPD and BoP in the test group were increased compared to the control. At 3 months, the clinical parameters of test group improved. All included subjects had decreased PPD, BoP, and PPD. CAL gain was observed in the control group with no significant changes in PPD, and BoP. The test group showed significantly shallower PPD and CAL than the control group.
O’Connell et al. (2008) [61] Brazil	Parallel, double blind, placebo control	30 individuals with DM2: - Test: 15 patients - Control: 15 patients	- Test: SRP + 100 mg doxycycline once a day for 14 days after an initial dose of 200 mg - Control: SRP + placebo	3 months	PPD reductions of 0.8 mm for the control group and 1.1 mm for the test group were noted. Mean PPD reduction of 0.9 mm and a mean CAL gain of 0.7 mm were evidenced. PPD decreased 23%, and BoP decreased 38% in the test group. PPD and CAL reductions were 0.8 and 0.5 mm in the control group. BoP was not significant between the groups (34.9% for the control and 42.4% for the test group) after 3 months. The test and control groups evidenced significant reductions in PI scores (22.2% and 23.8% for control and test groups).
Al-Zahrani et al. (2009) [51] Saudi Arabia	Single-masked, randomized, controlled trial	45 individuals with DM2: - SDD only group:15 - SRP + Doxy group: 15 patients - SRP + PDT group: 15 patients	- SRP + 100 mg doxycycline once daily for 13 days - SRP + PDT group: 0.01% methylene blue plus diode laser - Control: SRP	12 weeks	Statistically significant differences in mean PPD, CAL, PI, and BoP were found between baseline and 12 weeks post-treatment for all groups. The groups were balanced (no statistically significant differences) regarding the levels of PPD, CAL, PI, and BoP (*p* > 0.05). The mean CAL were similar between males and females (*p* > 0.05).
Engebretson and Hey-Hadavi (2011) [54] Colombia	Randomized placebo-controlled pilot clinical trial	45 individuals with DM2: - SDD group: 15 patients - ADD group: 15 patients - Control group: 15 patients	- SDD group: SRP + 20 mg doxycycline (2x/daily) - ADD group: 100 mg doxycycline for 14 days plus SRP - Control group: SRP + placebo	3 months	At one and three months’ follow-up, clinical periodontal parameters decreased in all groups. However, there were no statistical differences in clinical periodontal parameters between the groups. At three months, average HbA1c levels in the SDD group showed a 12.5% improvement.
Gaikwad et al. (2013) [56] India	Clinical study	50 individuals with DM2: - Test: 25 patients - Control: 25 patients	- Test: SRP + 100 mg doxycycline once a day for 15 days - Control: SRP	4 months	Both the test group and control group showed significant improvement in periodontal parameters over the experimental periods. The mean PPD for the test group and control group at baseline, 1, 2, 3, and 4 months were reduced. PPD between the two groups was significantly different at 4 months (2.67 ± 0.32 and 2.82 ± 0.21). There was a greater reduction in CAL in the test group (2.69 ± 0.42) than in the control group (2.99 ± 0.49). PI showed a statistically significant difference between the two groups. The differences in GI between the test and control group were not significant at any interval.
Botero et al. (2013) [53] Colombia	Randomized clinical trial	90 individuals with DM1 or DM2: - AZ-Sca group: 28 patients - PB-Sca group: 31 patients - AZ-Pro group: 31 patients	- AZ (test) group: SRP + 500 mg azithromycin once a day for 3 days - Placebo (control) group: SRP + placebo - AZ-Pro group: 500 mg azithromycin for 3 days + supragingival prophylaxis	9 months	Periodontal parameters were improved in the test and control group when compared to the AZ-Pro group. Mean BoP, PPD, and CAL were improved in the test and control group compared to the AZ-Pro group. However, a decrease in PPD (median Δ0.71 mm; *p* < 0.05) and number of sites with PPD > 4 mm was observed in the test as compared to the control group (median Δ0.39 mm) at 9 months. Improvement in CAL was similar between the test and control groups. The reduction in PPD was subtle in all groups after 9 months. The AZ-Pro group demonstrated no improvements in PPD and CAL in the evaluated periods.
Miranda et al. (2014) [60] Brazil	Randomized placebo-controlled clinical trial	56 individuals with DM2: - Test: 29 patients - Control: 27 patients	- Test: SRP+ 400 mg metronidazole + 500 mg amoxicillin three times a day for 14 days - Control: SRP + placebos	1 year	Clinical periodontal parameters were not different between groups at baseline. The test group showed lower mean PPD, mean number of sites with PPD ≥ 5 mm, decreased % of BoP, and suppuration compared to the control group. The test group presented a significant reduction in PPD and gain in CAL compared to placebo for moderate and deep sites. Reductions in the number of sites with PPD ≥ 5–6 mm were significantly greater in the test group compared to the control group.
Al-Nowaiser et al. (2014) [50] Saudi Arabia	Multi-center, randomized, parallel, single-blinded study	76 individuals with DM2: - Test: 38 patients - Control: 38 patients	- Test: SRP + 100 mg doxycycline once per day for 14 days, with an initial dose of 200 mg on the first day - Control: received no treatment other than oral hygiene	6 months	PPD was reduced by 0.93 mm in the test and 0.88 mm in the control group in the follow-up periods. CAL was reduced by 0.84 mm and by 0.96 mm in the period from baseline to 6 months in the test and control group, respectively. CAL was reduced by 0.39 mm in the test group and by 0.74 mm in the control group after 6 months’ follow-up. GI was not statistically significant between the test and control group after 1, 3, and 6 months after systemic doxycycline administration.
Hincapie et al. (2014) [55] Colombia	Randomized clinical trial	90 individuals with DM2: - Test group: 28 patients - Control group: 31 patients - AZPRO group: 31 patients	- Test group: SRP + 500 mg azithromycin once day × 3 days - Control group: SRP+ placebo - AZPRO group: supragingival prophylaxis + 500 mg azithromycin once a day for 3 days	9 months	Periodontal status in all the allocated groups was not significantly different between groups. An increase in CAL gain was observed in the test and control group, while no improvement was observed in the AZPRO group.
Tsalikis et al. (2014) [58] Greece	Randomized controlled clinical trial	66 individuals with DM2: - Test: 31 patients - Control: 35 patients	- Test: SRP + 200 mg doxycycline as an initial loading dose and 100 mg for 21 days - Control: SRP + placebo	6 months	No differences were noted between the groups for the clinical parameters evaluated. A statistically significant improvement was observed from baseline to 3 months in both groups. However, only patients in the test group showed differences between 3 and 6 months. The numbers of sites displaying PPD > 5 mm with BoP were significantly decreased in the test group in the 6-month period.
Tamashiro et al. (2016) [63] Brazil	Randomized controlled clinical trial	56 individuals with DM2: - Test: 29 patients - Control: 27 patients	- Test: SRP + 400 mg - metronidazole three times a day + 500 mg amoxicillin once a day both for 14 days - Control: SRP + placebo	2 years	The % of BoP, suppuration, and mean PPD were significantly lower in the test group at one and two years. The test group had significantly fewer sites with PPD ≥ 5 mm than the control at one and two years’ follow-up (control group = 14.7 ± 13.1, test group = 3.5 ± 3.4, *p* < 0.05). A greater reduction in mean PPD and CAL gain at moderate and deep sites were evidenced in the test group.
Vergnes et al. (2018) [28] France	Randomized controlled trial	76 individuals with DM1 or DM2: - Test: 34 patients - Control: 42 patients	- Test: SRP + chlorhexidine + 2 g amoxicillin once per day for 7 days and OHI - Control: SRP + chlorhexidine + OHI	3 months	Clinical periodontal status improved significantly after the treatment. PPD, CAL, and BoP improved significantly 3 months after SRP for type 1 and type 2 DM patients. No differences were noted in periodontal improvements according to diabetes duration and treatment (*p* > 0.05).
Das et al. (2019) [26] India	Randomized, parallel-arm, double-centered clinical trial	51 individuals with DM2: - Group 1: 17 patients - Group 2: 17 patients - Group 3: 17 patients	Group 1: OHI + SRP - Group 2: OHI + SRP + 100 mg doxycycline two times a day followed by once a day for 14 days - -Group 3: Did not receive treatment during study	3 months	Periodontal parameters were significantly higher in groups 1 and 2 compared to group 3 at 90 days. Mean PI decreased by 47.3% on day 90 (1.04 ± 0.28). The mean difference in PI in group 2 was 1.33 (53.20%). PI in group 3 increased by 0.06 (2.46%). GI in group 1 decreased by 45.0%. GI reduction in group 2 was 46.08%. GI in group 3 increased by 0.03 (2.46%). PPD in group 1 decreased by 24.67% (3.08 ± 0.30 and 2.32 ± 0.28). Mean PPD in group 2 decreased by 30.51%. PPD in group 3 increased by 0.10 from day 0 to 90 (2.53%). CAL reduced by 0.73 (19.26%) and 0.93 mm (24.73%) in groups 1 and 2, respectively. CAL in group 3 increased by 0.07 mm.
El-Makaky and Shalaby (2020) [27] Egypt	Randomized controlled trial	88 individuals with DM2: - Test: 44 patients - Control: 44 patients	- Test: SRP + OHI + 500 mg amoxicillin + 400 mg metronidazole (3x/day) for 14 days - Control: Treatment was carried out after the study	3 months	PI, PPD, BoP, and CAL were significantly decreased from baseline to 3 months in the test group. PI, PPD, BoP, and CAL were significantly increased from baseline to the end of the follow-up period in the control. Differences between the studied groups at 3 months were statistically significant.
Attia and Alblowi (2020) [52] Saudi Arabia		30 individuals with DM2: - Control: 15 patients - Test: 15 patients	- Control: SRP only - Test: SRP + 20 mg doxycycline twice a day for three months	3 months	The mean GI reduction in the control group was −0:73 ± 0:45 and −0:67 ± 0:55 at 1 and 3 months, respectively. The test group showed a decrease in GI by −0:97 ± 0:41 and − 1:27 ± 0:74 at 1 and 3 months, respectively. These differences were significant between the groups. PI within the control and test groups showed significant reductions between baseline, 1, and 3 months. Mean PPD was reduced throughout the study period in the control (−1:37 ± 0:61) and test group (−1:40 ± 0:50), without differences between them.
Cruz et al. (2021) [64] Brazil	Randomized controlled trial	58 individuals with DM2: - Test: 29 patients - Control: 29 patients	- Test: SRP + 400 mg metronidazole thrice a day + 500 mg amoxicillin for 14 days - Control: SRP + placebo	5 years	PI, BoP, PPD, CAL, and sites with PPD ≥ 5 mm in the test group showed decreased values at 5 years’ follow-up compared to baseline. The percentage of sites with BoP was reduced at 5 years in the control group. The mean number and percentage of sites with PPD≥ 5 mm were significantly lower in the test group than in the control group at 2 years, but not at 5 years.
Qureshi et al. (2021) [67] Pakistan	Three-arm randomized controlled trial	150 individuals with DM2: - Test 1: 50 patients - Test 2: 50 patients - Control: 50 patients	- Test 1: SRP + 400 mg metronidazole (3x/day for 10 days) + OHI - Test 2: SRP + OHI - Control group: OHI + SRP at the end of study	6 months	BoP, PPD, and CAL were significantly reduced after 6 months. No differences between the groups were observed. Mean PPD significantly increased in the control group, whereas BoP and mean CAL remained unaffected. There was a significant reduction observed in all periodontal variables in the test groups with respect to the control. However, there were no significant differences in periodontal variables between the two test groups.
Komatsu et al. (2022) [66] Japan	Randomized controlled trial	46 individuals with DM2: - Test: 24 patients - Control: 22 patients	- Test: SRP + 200 mg azithromycin once a day - Control: SRP	9 months	The control group exhibited no substantial alterations in comparison to the initial values across all the clinical parameters assessed (PPD, BoP, and GI). Conversely, the test group manifested noteworthy enhancements in clinical parameters in relation to the baseline measurements at 1, 3, 6, and 9 months (*p* < 0.001). Moreover, the test group demonstrated significant progress when compared to the control group at every time interval.
Mrag et al. (2023) [65] Egypt	Single-center cross-sectional study	125 individuals with DM2: - Test: 62 patients - Control: 63 patients	- Test: SRP + 500 mg amoxicillin and 500 mg metronidazole taken twice daily for 7 days consecutively - Control: SRP	3 months	A significant decrease in periodontal indices was observed in the test group. Compared to baseline, the control group did not show a significant decrease in periodontal indices. The test group showed a significant reduction in periodontal indices. Furthermore, pairwise comparison between the control group and test group showed a significant decrease in periodontal indices following antibiotic therapy.
Xu et al. (2024) [59] China	Short-term randomized controlled trial	49 individuals with DM2: - Test: 26 patients - Control: 23 patients	- Test: SRP + 500 mg amoxicillin and 200 mg metronidazole three times daily for 7 days - Control: SRP	3 months	Periodontal parameters improved significantly and similarly in both groups after treatment. The test group had more sites of improvement than the control group when the initial PPD was >6 mm. After treatment, the mean PPD decreased from 4.85 ± 0.97 mm to 3.74 ± 0.98 mm in the control group and from 4.86 ± 0.93 mm to 3.65 ± 0.96 mm in the test group. Additionally, the mean GI and PI values decreased and CAL significantly increased in both groups after treatment. When the initial PPD was >6 mm, the test group had more sites of improvement than the control group (698 sites [78.96%] vs. 545 sites [73.35%]).

Abbreviations: DM2—diabetes mellitus type 2; SRP—scaling and root planing; OHI—oral hygiene instructions; PPD—probing pocket depth; BoP—bleeding on probing; CAL—clinical attachment level; PI—plaque index; GI—gingival index.

**Table 3:** GRADE assessment for outcomes of two or more studies with methodological similarity.

Outcomes	Certainty Assessment						Summary of Results		
	Participants (Studies)	Risk of Bias	Inconsistency	Indirect Evidence	Imprecision	Overall Certainty of Evidence	Intervention	Comparator	Standard Mean Difference (CI95%)
Plaque index (SRP + doxycycline [1 month])	148 (3 RCTs)	Serious ^a^	Not serious	Not serious	Not serious	⨁⨁⨁◯ Moderate ^a^	75	73	SMD 0.3 SD (−0.29 to 0.36)
Probing depth (SRP + doxycycline [6 months])	134 (2 RCTs)	Not serious	Not serious	Not serious	Not serious	⨁⨁⨁⨁ High	66	68	SMD −0.27 SD (−0.61 to 0.07)
Bleeding on probing (SRP + doxycycline [6 months])	134 (2 RCTs)	Not serious	Not serious	Not serious	Not serious	⨁⨁⨁⨁ High	66	68	SMD −0.75 SD (−1.44 to −0.07)
Probing depth (SRP + azithromycin [6 months])	116 (2 RCTs)	Serious ^b^	Not serious	Not serious	Not serious	⨁⨁⨁◯ Moderate ^a^	57	59	SMD −0.47 SD (−0.84 to −0.1)
Probing depth (SRP + Metro/Amox [3 months])	193 (3 RCTs)	Not serious	Not serious	Not serious	Not serious	⨁⨁⨁⨁ High	99	94	SMD −0.35 SD (−0.91 to 0.22)
Bleeding on probing (SRP + Metro/Amox [3 months])	144 (2 RCTs)	Not serious	Not serious	Not serious	Not serious	⨁⨁⨁⨁ High	73	71	SMD −1.27 SD (−1.65 to −0.89)
Probing depth (SRP + minocycline—topical application route [3 months])	77 (2 RCTs)	Serious ^a^	Not serious	Not serious	Not serious	⨁⨁⨁◯ Moderate ^a^	46	31	SMD −0.37 SD (−1.15 to 0.41)
Bleeding on probing (SRP + minocycline—topical application route [3 months])	77 (2 RCTs)	Serious ^a^	Not serious	Not serious	Not serious	⨁⨁⨁◯ Moderate ^a^	46	31	SMD −0.93 SD (−1.42 to −0.44)
GRADE Working Group grades of evidence High quality: Further research is very unlikely to change our confidence in the estimate of effect. Moderate quality: Further research is likely to have an important impact on our confidence in the estimate of effect and may change the estimate Low quality: Further research is very likely to have an important impact on our confidence in the estimate of effect and is likely to change the estimate. Very low quality: We are very uncertain about the estimate.									
Explanations a. Methodological limitations related to allocation concealment, blinding processes, and incomplete outcomes. b. Methodological limitations related to blinding of outcome assessment and incomplete outcomes.									

Legend: BoP, bleeding on probing; CI: confidence interval; PD, probing depth; PI, plaque index; RCTs, randomized controlled trials; SMD: standardized mean difference. Meta-analyses with *p* ≤ 0.1 in the χ^2^ and I^2^ ≥ 75% were not included in the GRADE analysis. Regarding the certainty of the evidence, this is high, as expressed in the table by means of “⨁” figures (⨁◯◯◯ very low; ⨁⨁◯◯ low; ⨁⨁⨁◯ moderate; and ⨁⨁⨁⨁ high).

## Data Availability

Data generated in this research project can be accessed by contacting the last author of this paper via email. They are stored electronically as Excel worksheets.
